# COVID-19: Its Impact on Delayed Management of Pre-established Chronic Conditions

**DOI:** 10.7759/cureus.44667

**Published:** 2023-09-04

**Authors:** Camila A Villacreses, Andrew B Herson, Davong D Phrathep, Chigozie Igbonagwam, Sean A Briceno, Hamaad A Khan, Zain Barnouti

**Affiliations:** 1 Podiatry, Lake Erie College of Osteopathic Medicine, Jacksonville, USA; 2 Podiatry, St. Vincent's Medical Center, Jacksonville, USA

**Keywords:** necrotizing fascitis, diabetes, covid 19, chronic venous insufficiency (cvi), chronic venous leg ulcers

## Abstract

Chronic venous insufficiency (CVI) is a common condition affecting the venous system, typically arising in the setting of increased venous pressure and impaired blood return secondary to weakened valves or damaged veins. Diabetes mellitus causes impaired circulation, neuropathy, impaired healing, and increased susceptibility to infection. Because diabetes and CVI are interconnected, ulcerations can progress to necrotizing fasciitis if not treated promptly and appropriately. The coronavirus disease 2019 (COVID-19) pandemic has further complicated patient care and is a potential risk for complications and delays in the management of time-sensitive conditions like necrotizing fasciitis. Here, we present a case study highlighting the impact of COVID-19 on the delayed management of necrotizing fasciitis in a 51-year-old male with multiple comorbidities.

## Introduction

Chronic venous insufficiency (CVI) describes a condition that commonly affects the venous system of the lower extremities [1]. The peripheral venous system functions as a reservoir to store blood and the patency of the vessels and the muscles surrounding the vasculature enable blood to flow properly and return to the heart via one-way valves [2]. Venous pathology develops when venous pressure is increased, and the return of blood is impaired [3].

Venous insufficiency can result from weakened or damaged veins within the valves, resulting in inadequate unidirectional blood flow in the body, especially the lower extremities. When the venous valves fail to function adequately, patients typically present with pain, swelling, and skin changes. The more serious consequences of venous insufficiency are ulcers, which have a poor prognosis if there is a delay in healing and if recurrent ulcerations occur [1]. Foot ulcers in diabetic patients are common and result because of poor circulation, neuropathy, and impaired healing [4]. Thus, if left untreated or if there is a delay in treatment, ulcers can potentially result in a rapidly progressive, life-threatening soft tissue infection called necrotizing fasciitis.

Diabetic foot ulcers are a major complication of diabetes mellitus. Risk factors include age, previous ulceration, and diabetic polyneuropathy with neuropathy accounting for 50% of cases of diabetic foot ulcers [5]. Thirty-five percent of cases occur due to a combination of neuropathy and angiopathy while 15% are due to peripheral arterial disease [5]. Diabetic neuropathy results in decreased vibratory sensation along with paresthesia which consequently reduces the sensation of pain resulting in an increased risk for trauma [5].

Necrotizing fasciitis is characterized by rapidly progressing inflammation and necrosis of the subcutaneous fascial tissues with or without adjacent muscle involvement [6]. With potential to be either polymicrobial or monomicrobial, necrotizing fasciitis can present as an emergency infectious disease. It may require prompt surgical debridement, intravenous fluids, antibiotics, analgesia, electrolyte management, and grafting in severe cases [7].

In a recent study, 41% of adults in the United States delayed or avoided medical care due to coronavirus disease 2019 (COVID-19) concerns [8]. Because our patient contracted COVID-19, he was unable to attend his scheduled wound care appointments for three weeks. Our report highlights the impact COVID-19 had on the management of necrotizing fasciitis in a 51-year-old male with multiple comorbidities, including diabetes mellitus

## Case presentation

A 51-year-old male with a past medical history of insulin-dependent diabetes mellitus, hypertension, dyslipidemia, hypothyroidism, and diabetic neuropathy presented to the emergency department with complaints of fever, chills, poor appetite, and generally feeling unwell for the past five days. He reported a history of a right foot diabetic ulcer that was initially cared for by his podiatrist, and believed the wound was the culprit of his current state of health. The patient also stated that for the past three weeks he was unable to go to his scheduled wound care appointments due to contracting COVID-19. He reported having nausea, vomiting, body aches, and diarrhea which had also been present for the past four days.

Initial vital signs on presentation were heart rate of 104 bpm (peripheral), blood pressure of 111/66 mmHg, an oxygen saturation (SpO2) of 97% and a temperature of 100.4 degrees Fahrenheit. Physical examination was unremarkable except for a right foot wrapped with dressing. Upon removal of the dressing, plantar and dorsal forefoot ulcers were with surrounding cellulitis. The superficial ulcer had irregular edges measuring 8.1 x 5.1 x 2 cm. There was also heavy sloughing of fibrotic and necrotic tissue in the base. The wound was malodorous with erythema and swelling around the edges. Some exposed tendon was also observed.

Laboratory tests included a complete blood count (CBC) and a comprehensive metabolic panel (CMP). The CBC revealed chronic anemia (Table 1). The CMP revealed an elevated creatinine (Table 2).

**Table 1:** Complete Blood Count L: Liter; g/dL: grams per deciliter; μm3: cubic micrometers; pg/cell: picograms per cell; Hb/cell: hemoglobin per cell; mm3: cubic millimeters; fL: femtoliters.

	Laboratory Values	Reference Range
White blood cell count	15.6	4.5 to 11.0 × 10^9^/L
Red blood cell count	4.58	4.3-5.9 x 10^12^/L
Hemoglobin	12.7	13.5-17.5 g/dL
Hematocrit	38.0	41%-53%
Mean Corpuscular Volume	82.9	80-100 μm^3^
Mean Corpuscular Hemoglobin	27.6	25-35 pg/cell
Mean Corpuscular Hemoglobin Concentration	33.3	31%-36% Hb/cell
Red Blood Cell Distribution Width	14.3	12-15%
Platelet Count	166	150,000-400,000 mm^3^
Nucleated Red Blood Cell Count, percentage (NRBC %)	0	0%

**Table 2:** Comprehensive Metabolic Panel BUN: blood urea nitrogen; eGFR: estimated glomerular filtration rate; A/G ratio: albumin to globulin ratio; mg/dL: milligrams per deciliter; mL/min/1.73m2: milliliters per minute per 1.73 meters squared; mmol/L: millimoles per liter; g/dL: grams per deciliter; U/L: units per liter.

	Laboratory Value	Reference Range
Sodium	131	135-145 mmol/L
Potassium	3.7	3.5-5 mmol/L
Chloride	96	95-105 mmol/L
Carbon Dioxide	23	22-32 mmol/L
Anion Gap	12	4 to 12 mmol/L
BUN	25	7-25 mg/dL
Creatinine	1.4	0.7-1.5 mg/dL
Glucose	155	70-100 mg/dL
Calcium	9.2	8.5-10.5 mg/dL
Aspartate Transaminase	15	10-35 U/L
Alanine Transaminase	17	0-31 U/L
Alkaline Phosphatase	77	25-125 U/L
Protein, Total	6.8	6.5-8.1 g/dL
Albumin	3.2	3.5-5.0 g/dL
A/G Ratio	0.9	0.8-2.0 g/dL
Bilirubin, Total	1.3	0.0-1.2 mg/dL
eGFR	61	>60 mL/min/1.73m^2^

The creatinine was higher than his baseline of 1.1. This was attributed to the antibiotics he was taking for the diabetic foot ulcer prior to admission.

**Figure 1:**
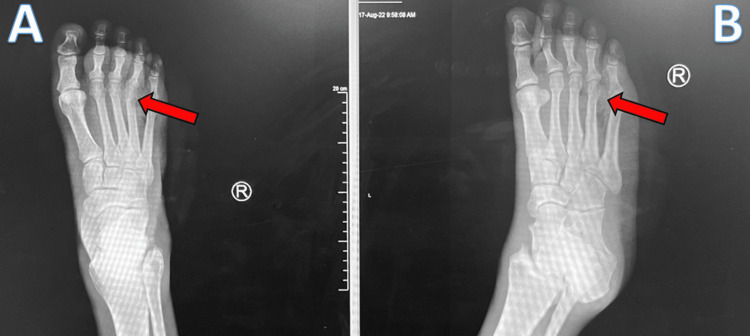
Anterior and Lateral X-Ray Images of Right Foot Anterior (A) and lateral (B) view of right foot demonstrating extensive subcutaneous gas along the fibular and dorsal aspect of the forefoot (red arrows).

X-ray imaging of his right foot showed extensive subcutaneous gas present along the fibular and dorsal aspect or the forefoot, which was likely indicative of an infection with gas-forming organisms/open wound (Figure 1).

**Figure 2:**
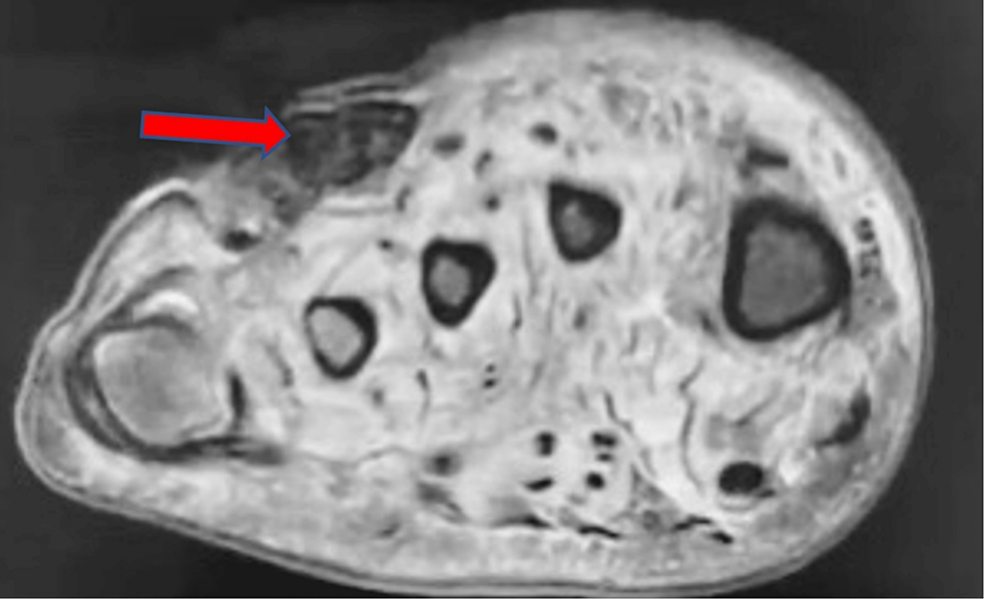
Axial MRI Without Contrast of the Right Foot Axial MRI Without Contrast of the Right Foot showing ulcers in the dorsum of the forefoot with some tracking of the subcutaneous air communicating with the skin lesions (red arrow).

**Figure 3:**
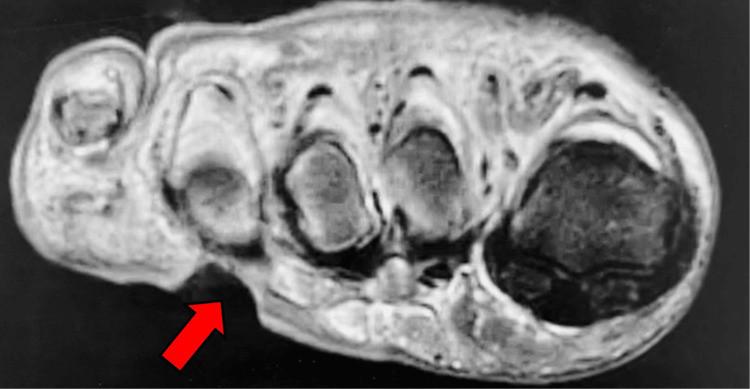
Axial MRI Without Contrast of the Right Foot Lower axial plane of MRI shows ulceration of the plantar soft tissues near the fourth metatarsal head (red arrow).

**Figure 4:**
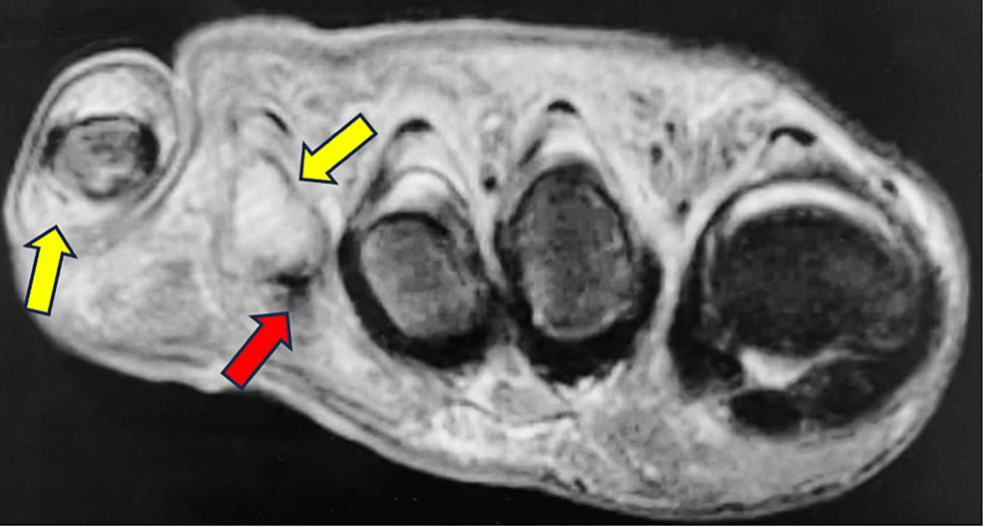
Axial MRI Without Contrast of the Right Foot An abnormal marrow signal is seen in the mid to distal fourth metatarsal/proximal phalanx, fifth metatarsal head/proximal phalanx compatible with osteomyelitis (yellow arrow). A periosteal abscess is also present on the fourth metatarsal head (red arrow).

MRI without contrast of the right foot was positive for osteomyelitis involving the mid to distal fourth metatarsal and fourth proximal phalanx as well as in the fifth metatarsal head and the fifth proximal phalanx (Figure 2). Different views of the MRI also indicated ulceration of the soft tissues on the plantar aspect of the foot (Figure 3). Additionally, MRI also showed abnormal marrow signaling consistent with osteomyelitis and a periosteal abscess on the fourth metatarsal head (Figure 4).

A wound culture was obtained on his second day of hospitalization. Infectious disease and podiatry were consulted. The patient was diagnosed with necrotizing fasciitis and osteomyelitis, and was started on vancomycin, Zosyn, and clindamycin.

Subsequent wound cultures were positive for multi-bacterial infection consisting of Streptococcus agalactia, Klebsiella pneumoniae, and coryne-like diphtheroid. His antibiotic treatment was adjusted to metronidazole according to culture and sensitivities.

Once the patient’s medical condition was stable, he subsequently underwent various surgical procedures including a partial bone resection/distal metatarsal osteotomy of the right fourth metatarsal, partial bone resection/base of the proximal phalanx osteotomy of the right fourth toe, and a capsulotomy of the right third and right fifth metatarsophalangeal joint (MPJ). Additionally, a deep incision and drainage (I&D) of the mid-right foot and an excisional debridement including muscle, tendon, and fascia measuring around 10x5 cm on the right foot.

Disposition of the patient included a more rigid diabetic control to avoid future complications of diabetic foot ulcers. He would also receive physical therapy to regain strength in his right foot, as well as a regimen of daily wound care. He also had scheduled debridement with subsequent synthetic skin grafts and proper wound dressing. Due to the management of his infection and the surgical procedures he underwent, he had an excellent prognosis and managed a full recovery of his wound.

## Discussion

CVI is defined by changes in the physiological function of the venous system, primarily of the lower extremities, that lead to skin changes like ulcers and telangiectasias, varicose veins, edema and changes in extremity pressure. CVI is typically caused by a combination of venous hypertension, venous valve insufficiency, and subsequent stasis leading to vascular and integumentary breakdown. Risk factors for CVI include advanced age, sex, history of deep vein thrombosis (DVT), sedentary lifestyle, use of oral contraceptives, leg injury, and systemic hypertension among others [9]. The treatment goals for CVI are to reduce discomfort and edema, stabilize skin appearance, remove painful varicose veins, and heal ulcers. Compression bandages may be used to help with ulcer healing. Patients who fail to respond to compressive bandages may need surgical intervention [10]. CVI can become even more complex when presented alongside sequel of diabetes, such as diabetic foot ulcers. 

The primary therapy for intervention of diabetic foot ulcers and their complications is prevention [11]. Studies have shown that the utilization of podiatry services in patient care is associated with decreased lower extremity amputation and reduction in hospital admission rates [11]. Clinical examination can be used to classify risk stratification of complications [11]. Patient education on basic foot care, appropriate footwear and wound care also plays a significant role in delaying the onset of recurrent foot ulcers [11].

 In this case presentation, the patient was compliant with wound care appointments and follow-up; however, after developing COVID-19, he was unable to continue his wound care appointments. This inability to continue his wound care due to COVID-19 likely contributed to the worsening of his CVI and the development of his necrotizing fasciitis. Luckily, the patient was able to obtain sufficient medical care to treat his necrotizing fasciitis and managed a full recovery of his wounds.

As demonstrated by this case, delaying medical care can exacerbate existing injuries and increase risks associated with preventable pathologies. In medical conditions that require constant care by clinicians, such as the CVI presented in this case, any interruption in timely medical attention can cause the insult to progress and become increasingly complex.

The COVID-19 pandemic was an enormous hurdle for patients and clinicians alike to accommodate for safe interaction while still ensuring patients received adequate care. Literature review has found a high correlation on the disproportionally negative impact that COVID-19 has had on individuals living with chronic medical illnesses. Those with pre-existing cardiovascular disease, such as hypertension and coronary heart disease, are at a greater risk of developing severe and potentially fatal COVID-19 [12]. While those who are obese are at a higher risk of requiring more complex and aggressive ICU management. The increased severity of COVID-19 has been further acknowledged in diabetes, as it can lead to hyperglycemia, altered immune function, sub-optimal glycemic control while hospitalized, and prothrombotic as well as pro-inflammatory states [12]. The social isolation that accompanies the management of COVID-19, takes a toll on all patients, but even more so on those with pre-existing mental health disorders. 

A cross-sectional study analyzed the associations between delayed medical care and underlying health, demographic, and regional factors in the United States during the COVID-19 pandemic [8]. Some of these factors included the presence of pre-existing conditions, sociodemographic and health characteristics, and delayed medical care [8]. People with lower household income experienced higher levels of financial strain throughout the pandemic. Their financial strain could have an additional impact on their ability to receive medical care and thus further delay the healthcare they needed. The study also found that people with poorer overall health were more likely to delay medical care [8]. 

In our case, comorbidities and COVID-19 status impacted our patient’s ability to continuously receive the previously established proper care for his CVI, which in turn led to further worsening of his condition. 

## Conclusions

This case highlights the not-so-readily-seen consequences that the contraction of COVID-19 has on pre-established medical conditions, either due to financial strain or delayed treatment due to the period of isolation COVID-19-positive patients must adhere to. The patient presented had successful clinical management despite the delay in treatment, secondary to his contraction of COVID-19, which led to him developing necrotizing fasciitis from a previous foot ulcer that was being properly managed. As such, this report navigated the polymicrobial nature of necrotizing fasciitis, the administration of proper antibiotics, and prompt use of necessary surgical intervention as well as a post-operative treatment plan to avoid complete amputation of the patient’s foot.
